# Incentivizing news consumption on social media platforms using large language models and realistic bot accounts

**DOI:** 10.1093/pnasnexus/pgae368

**Published:** 2024-08-23

**Authors:** Hadi Askari, Anshuman Chhabra, Bernhard Clemm von Hohenberg, Michael Heseltine, Magdalena Wojcieszak

**Affiliations:** Department of Computer Science, University of California, Davis, USA; Department of Computer Science and Engineering, University of South Florida, Tampa, USA; GESIS—Leibniz-Institute for the Social Sciences, Cologne, Germany; Amsterdam School for Communication Research, University of Amsterdam, Amsterdam, The Netherlands; Amsterdam School for Communication Research, University of Amsterdam, Amsterdam, The Netherlands; Department of Communication, University of California, Davis, USA

**Keywords:** social media, news engagement, bots, polarization, news avoidance

## Abstract

Polarization, misinformation, declining trust, and wavering support for democratic norms are pressing threats to the US Exposure to verified and balanced news may make citizens more resilient to these threats. This project examines how to enhance users’ exposure to and engagement with verified and ideologically balanced news in an ecologically valid setting. We rely on a 2-week long field experiment on 28,457 Twitter users. We created 28 bots utilizing GPT-2 that replied to users tweeting about sports, entertainment, or lifestyle with a contextual reply containing a URL to the topic-relevant section of a verified and ideologically balanced news organization and an encouragement to follow its Twitter account. To test differential effects by gender of the bots, the treated users were randomly assigned to receive responses by bots presented as female or male. We examine whether our intervention enhances the following of news media organizations, sharing and liking of news content (determined by our extensive list of news media outlets), tweeting about politics, and liking of political content (determined using our fine-tuned RoBERTa NLP transformer-based model). Although the treated users followed more news accounts and the users in the female bot treatment liked more news content than the control, these results were small in magnitude and confined to the already politically interested users, as indicated by their pretreatment tweeting about politics. In addition, the effects on liking and posting political content were uniformly null. These findings have implications for social media and news organizations and offer directions for pro-social computational interventions on platforms.

**Significance Statement**

Most citizens do not consume news and public affairs on social media platforms. Because news exposure can make citizens more resilient to various democratic threats, this project incentivized users’ engagement with credible and ideologically balanced news. We created 28 Large Language Model-trained bots that responded to users’ tweets about entertainment, sports, or lifestyle with a contextual response, encouragement to follow a news outlet, and a link to an interest-relevant section of the outlet. Our 2-week field experiment on 28,457 Twitter users tested if responses by female or male bots increased users’ following and posting of news and liking and posting about politics. We find small and largely insignificant effects that are mostly confined to the already politically engaged users.

## Introduction

Polarization, declining trust, and wavering support for democratic norms are pressing threats to the US. Observers often blame social media platforms for these problems, worrying about misinformation, echo chambers, and algorithmic radicalization (1–4). Evidence to support these worries, however, is limited. Few people inhabit echo chambers (5–7), encounter or are affected by misinformation (8–10), or are put in extreme rabbit holes (11, 12).

We argue that the problem is less that people consume bad political information, but that most people do not consume any at all. News and politics constitute a small fraction of people’s information diets on social media. News makes up only 1.4% of Facebook’s News Feed (13, 14), the majority of Twitter users do not follow any politicians, journalists, or news organizations (6), and only about 1 in 300 outbound clicks from social media are to substantive news (15).^a^

This under-consumption of news—and the consequently low levels of political knowledge among the electorate—have important implications (19). Low-information voters are likely to withdraw from politics, vulnerable to making irrational vote choices (20–22), and easily swung by irrelevant stimuli, emotional appeals, and populist rhetoric in the political environment (23). If sizable, these voters can swing elections (21, 24). In turn, exposure to verified and ideologically balanced news creates an informed public and leads to more stable political attitudes, lower susceptibility to misinformation, greater acceptance of democratic norms, and voting in accordance with one’s interests (19, 25–28). Some note that news exposure is the key predictor of political knowledge, seen as “a demonstrably critical foundation for good citizenship” (25).^b^

Given these benefits, it is of considerable interest to promote the consumption of factual news on social media. This project aims to incentivize Twitter (current X) users to engage with *verified and ideologically balanced* public affairs information.^c^ We conduct a large-scale field experiment on 28,457 US-based Twitter users who mostly engage with non-political topics, i.e. tweet about lifestyle, entertainment, and sports (see Supplementary Material S1 for details on the sample and its selection). We reach those users through their non-political interests and direct them to interest-relevant parts of news outlets with the expectation that this will encourage those users to access and follow verified and balanced news on social media. ^d^

Toward this end, we rely on NLP-trained bots to reply contextually and in real time to original nonpolitical tweets of these active users over 2 weeks. Our GPT-2-generated responses include a relevant reply, specific to the content of the original tweet (e.g. “He’s the best player in the league” as a response to a tweet about a baseball pitcher, see Supplementary Material S2). In addition and serving as the core treatment, the responses include two core hardcoded elements: a link to topic-relevant nonpolitical section of a verified and ideologically balanced news media organization and an encouragement for the users to follow the Twitter account of that organization. To identify verified and balanced news outlets, we apply validated expert metrics based on human coding from Ad Fontes, selecting only the outlets that score high on reliability and low on partisan bias (see Supplementary Material S3A for details on outlet selection and Supplementary Material S2 for the hardcoded elements).

Our sample was randomly assigned to one of two treatment groups, receiving responses from bots presented as either male or female for 2 weeks, or a control group. We rely on our extensive curated list of US news organizations (see Supplementary Material S3) and a validated BERT-based classifier that identifies users’ tweets about politics (see Supplementary Material S4) to test whether users (i) follow news accounts on our list, (ii) retweet content from news media organizations, (iii) tweet or retweet political content, (iv) like content from news media, and (v) like political content.

We find that encouragement to follow news through our tailored NLP-based responses had some promising, yet limited, effects. It encouraged the users to follow more news outlets and encouraged those who received comments from the female bot to like more news content on social media. These effects, however, were small in magnitude and the treatment had no effects on the other outcomes analyzed, i.e. (re)tweeting news content and tweeting about or liking posts about politics. The increases in the liking of news media content, moreover, were confined to those with high initial levels of political interest, as indicated by previous tweeting about politics, suggesting reinforcement of pre-existing engagement among those already engaged (33, 34). Also, the effects on news media content liking were especially pronounced for the users who were tweeting about sports, with the effects among those who tweeted about entertainment or lifestyle being statistically insignificant due to the decreased sample sizes in these two topic categories.

This project advances past work in several key ways. First, we address the problem of low news use and news avoidance (35–37). The overwhelming majority of social media users go online for entertainment, not news or politics (13, 38–42). Because information exposure on platforms is primarily driven by recommender algorithms that make automated decisions on what content to display based on the user’s past behavior and inferred interests (43), those users are mostly recommended contents about sports, movies, or celebrities. These personalized recommendations ultimately create closed loops of entertainment consumption and narrow information repertoires (38, 44). Our intervention aims to break this feedback loop. Following news organizations and clicking on news links embedded in the responses from our bots puts public affairs information in the users’ inventory. That is, posts from the followed accounts would automatically display in the users’ feed, increasing the likelihood that the users see and engage with this information (45). In addition, following news accounts signals to the algorithms that the user is interested in news and politics, thus generating subsequent recommendations to public affairs content (38). In short, our intervention overcomes news non-use by increasing the chances that users easily encounter publicly relevant content in their social media ecosystem and creating positive feedback loops, in which algorithms recommend more news and politics.^e^

Second, we reach those users through their non-political interests, an approach found effective in the work on soft news. Research on soft news or “infotainment” suggests that programs that discuss cooking or celebrities, but also mention current affairs, attract viewers whose primary motivation is not politics, but who nevertheless learn about current affairs and become more politically active (48–53). Accordingly, we engage users interested in sports, entertainment, and lifestyle by connecting these topics with news and directing users to news outlets that offer both hard news and softer news about sports, movies, cooking, or wellness. Starting from citizens’ non-political interests and facilitating easy access to topically relevant content in primarily hard news outlets (e.g. the lifestyle or sports sections of ABC News), we aim to enhance users’ interest in news and politics and sustainably increase their exposure to factual news. Social media platforms act as an intermediary to news organizations (54, 55) and so encouraging users to follow news accounts and to visit news sites through links embedded in posts may serve as a gateway to hard news consumption (56).

Third, differential engagement may occur based on who is sharing news with the users and also who the users are. Our experiment systematically varied the presented gender of the bot, whether female or male, expecting that the effects from our treatment would be stronger for male sources than from female sources. In general, news and politics are seen as male-dominated spaces (57, 58), which has important implications for how females are received in these fields. Females are perceived as less credible than males in political ads, especially in the contexts of more masculine issues (59, 60), women are less likely to be quoted as expert sources than males (61), male journalists engage almost exclusively with their male colleagues on Twitter (62), and female media figures receive more toxic, abusive, and hostile responses compared to their male counterparts in general (63–65) and especially in political (66) and science (67) contexts. Therefore, social media users may be less inclined to follow suggestions or open links from female sources or to see them as a news source worth interacting with.

In addition, there may be heterogeneous treatment effects by users’ political interest. On the one hand, politically disinterested individuals may gain more in terms of knowledge, engagement, or subsequent news seeking from soft news programming (50, 56) and from inadvertently seeing politics online (68). That is, the equalizing hypothesis predicts that encountering politics on social media could enhance knowledge or participation especially among those with low political interest (33). In our study, seeing comments with links to interest-relevant sections of news websites may attract the attention of low-interest individuals when these comments connect to their non-political interests. On the other hand, inadvertent exposure to news and politics may reinforce existing gaps in prior political interest (33, 69) and create reinforcing spirals such that those already politically inclined engage more with the encountered news, which further enhances their political interest and involvement (70). This is because the more politically interested individuals may be more likely to see recommendations to news as relevant and to process them more carefully (33, 71). In turn, those with very low political interest may react negatively to unwanted political content and reject the recommendations to engage with news (33). In short, individuals may be responsive to social media nudges but the source (gender) of these nudges as well as users’ prior posting about current affairs may be important factors influencing these results.

## Data and measurement

**Fig. 1.**
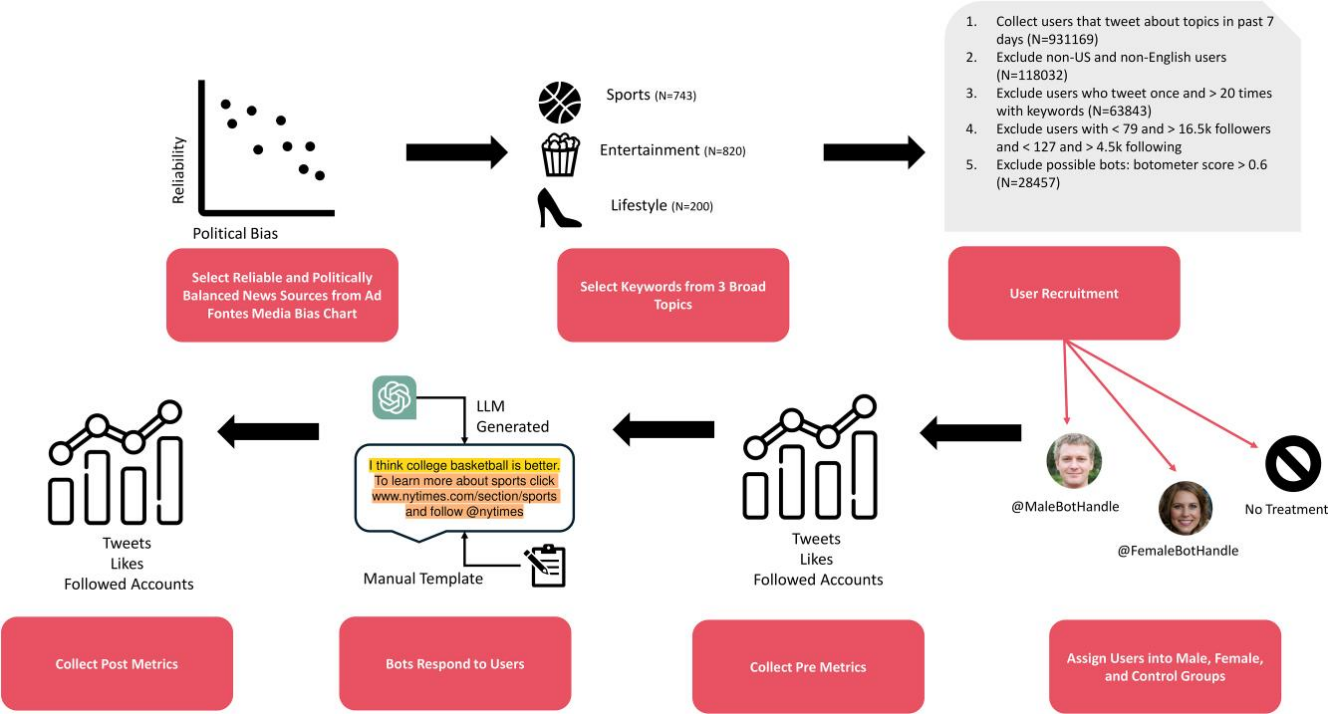
Overview of the experiment design.

The overview of the design is presented in Fig. 1. We identified US-based Twitter users who actively tweeted about one of three non-political topics: sports, entertainment, and lifestyle, across a 1 week period in September 2022. To do this, we created a list of 1,763 keywords generated using word embeddings and manual additions (e.g. current movies and television series, athletes, brands; see Supplementary Material S1A for details; keywords broken down by topic are available at Github). We collected our initial user base by scraping the user IDs of all Twitter users who tweeted our keywords at least once in a 7 day period, with location and language filters to ensure that only users based in the US and tweeting in English were included (N=118,032). We then excluded those who tweeted only once during the 7 day period, as these infrequent users were relatively unlikely to be active during the treatment period. To minimize the chances that power users or administrative accounts (e.g. celebrities, brands, or organizations) are represented in our sample, we also excluded users who tweeted more than 20 times (N remaining = 63,843) and those who were in the top 10th and 90th percentiles of followers and followees (i.e. those who had fewer than 79 or more than 16,500 followers and those who followed fewer than 127 or more than 4,500 accounts). Finally, we removed all users with a botometer score of more than 0.60 to minimize the inclusion of bots (72). This resulted in a final sample of 28,457 active nonbot US-based users known to tweet about the three nonpolitical topics more than once a week.

These users were randomly assigned to one of three groups: a control, a male bot treatment, or a female bot treatment. Randomization was successful on a range of account level metrics (the total number of followed accounts, total number of followers, total posts, and total likes) as well as central pretreatment metrics (the number of news accounts followed, the number of recent likes of news posts, the number of (re)tweets of posts from news accounts), ensuring balance across groups in terms of existing engagement with news media. All pre-experiment metrics were collected 1 week prior to the start of the experiment using the Twitter API. Supplementary Material S1B details the assignment and randomization.

**Fig. 2.**
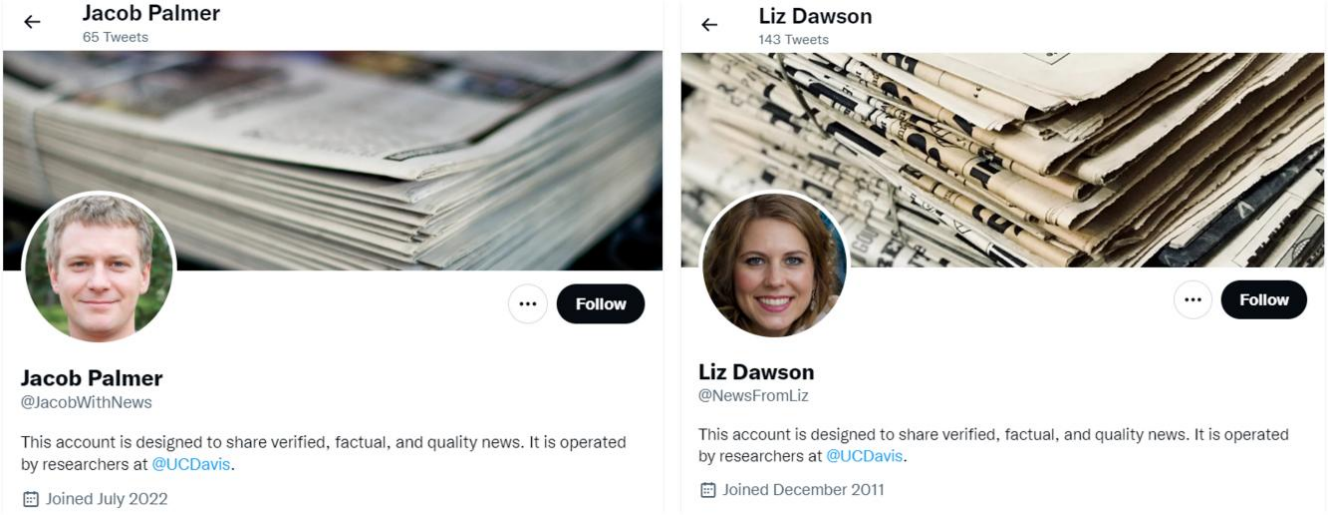
Sample male and female bot accounts.

We created 28 bots utilizing GPT-2 to contextually reply to the users in the sample (14 bot accounts for male and 14 for female treatment group). The bots were designed to be realistic and substantively similar, with gender-definable headshot pictures, gender-identifiable names, and a history of news-related content in their feed (Fig. 2 shows two examples, see Supplementary Material S1D for additional details on bot creation).^f^

To generate responses to the treated users, we leveraged GPT-2 models (73).^g^ This model was fine-tuned by Microsoft on Reddit comments and was specifically designed to be conversational in nature. This ensured that the responses were contextually relevant and applicable to the original tweet sent by a user. This contextual nature of the responses, i.e. the fact that each was different and adapted to the original user’s tweet, also reduced the likelihood that they were considered spam and banned by Twitter. Before sending the Tweets to the GPT-2 model, we removed all URLs and special characters. Additionally, we discarded the GPT-2 response if (i) it contained language pertaining to Reddit (such as upvote, subreddit, etc.), (ii) kept on repeating the same text, and (iii) used profanity. In cases where responses were discarded, the contextual text was replaced by a randomly selected hardcoded template response. In addition to the GPT-2 based reply to each user’s tweet, we hardcoded two elements into the response. We encouraged users to follow a news media organization (e.g. “follow @wsj” or “follow @nyt”) and to visit a link to a relevant sub-section of a verified and ideologically balanced news source (e.g. an entertainment/sports/lifestyle section of the Wall Street Journal or the New York Times). Supplementary Material S2 offers details on this process.

To ascertain that our intervention directed users to verified and balanced news outlets, we applied validated expert metrics. We compiled a list of reliable and ideologically balanced news sources from Ad Fontes (74). Ad Fontes relies on manually labeled articles, radio, TV, and videos (episodes) from numerous news sources. Each episode is rated by trained human coders and scores are assigned for reliability (from “contains inaccurate/ fabricated information” to “original fact reporting”) and ideological bias (from “most extreme left” to “most extreme right”). We selected news outlets with a reliability score higher than 40 and a bias score between −18 and 18 (see Supplementary Material S3A for details). These outlets, their scores, and the URLs to the relevant sub-sections recommended to the users are shown in Supplementary Material S3A.^h^

The experiment was fielded between 1/19/2023 and 2/3/2023. Every 8 hours we scraped the timelines of all users. Tweets matching one of our topic keywords would then receive an automated reply from an assigned bot account, which contextually and dynamically matched the reply to the original tweet of a user. Each response also encouraged the user to stay up to date with the news and visit a link to a topic-relevant sub-section of a news source from our list, as aforementioned. We limited the number of responses to one per day, so as to ensure that the users who tweet using our topic keywords multiple times a day would not be irritated or see our responses as spam. The scraping and response cycle ran continuously for 2 weeks. After this time period, the treatment was terminated.

We collected 3 pretreatment and post-treatment behavioral metrics from all the users: the followed accounts (pre N=6,536,692, post N=17,286,211),^i^, tweets or retweets (pre N=2,285,401, post N=2,201,009), and likes (pre N=2,927,951, post N2,846,354).

To examine if our intervention increased engagement with news and politics on Twitter, we collected post-treatment metrics 1 week after the termination of the treatments, contrasting these results with pretreatment collections of the same measures (based on the prior 100 (re)tweets and likes from a user before the treatment period). We first assessed whether users followed news organizations from the Ad Fontes list or any additional news outlets. To measure whether users (re)tweeted or liked news content, we used our extensive curated list of over 5,400 News Media organizations. The details on the creation of the overall list are presented in Supplementary Material S4B, and the list is made publicly available on Github. We identified Twitter handles for 5,341 news organizations from the overall list and identified each user’s likes and (re)tweets from these news outlets.

To measure whether users (re)tweeted or liked political content on Twitter, we developed a fine-tuned RoBERTa classifier of political content (76). We conceptualize “politics” rather broadly: tweets considered as political include references to political figures, policies, elections, and specific political events *as well as* issues such as climate change, immigration, healthcare, gun control, sexual assault, racial, gender, sexual, ethnic, and religious minorities, the regulation of large tech companies, and crimes involving guns. The classifier was specifically trained on social media data and identifies content about politics with high accuracy (accuracy=0.93, precision=0.92, recall=0.91, F1=0.915). Supplementary Material S4C shows the details on model training, fine-tuning, performance, and validation. For each user in our sample, we identified all the instances of liking and tweeting political content on the platform.

We also measure to what extent the treated users interacted with our bots, by checking whether the users replied to the responses generated by the bots. Lastly, we evaluate the sentiment of these user replies using a RoBERTa-base model trained on 124M tweets from January 2018 to December 2021, and fine-tuned for sentiment analysis with the TweetEval benchmark (77, 78). We collected a total of 241 (99 male and 142 female) responses and examined their sentiment. See Supplementary Material S4E for details.

These measures together comprehensively portray users’ pre- and post-treatment posting about and engagement with both news and politics. Treatment effects are examined as the difference in pre- and post-treatment measures for both the female and male treatment groups compared to the control group. For the difference in news media accounts followed, this measure is taken as a simple integer value. For the difference in the liking and (re)tweeting of news and political contents, these pre- and post-differences are measured as the change in the percentage of (re)tweets and likes from news media accounts or about politics, based on user activity measured specifically in the pretreatment period and the 1 week after the treatment period.

## Results

### Descriptives

We first describe the pre-treatment following and engagement metrics among our sample to offer a baseline. On average, our users followed 14 news accounts (both male and female treatment groups) prior to our treatment. The levels of users’ engagement with news content, namely the pre-treatment proportion of likes on and (re)tweets of posts coming from one of the 5,341 news organizations relative to all likes and (re)tweets a user had in the pre-treatment collection period, were very low among our sample.^j^ For the liking of news media content, this figure was 0.8% on average, and for (re)tweeting news media content, this figure was 0.4%. In short, engagement with news media content was a very infrequent activity. The liking of *political* content was more frequent, likely due to our rather broad conceptualization of what constitutes political content (i.e. not only traditionally hard news such as the election, political parties, the economy, etc. but also social issues, such as race, immigration, abortion, etc.; see Supplementary Material S4C for the details). Likes on political content constituted just under 12% of all likes among our users. Similarly, the percentage of political (re)tweets was around 11.5% across the user groups.

**Fig. 3.**
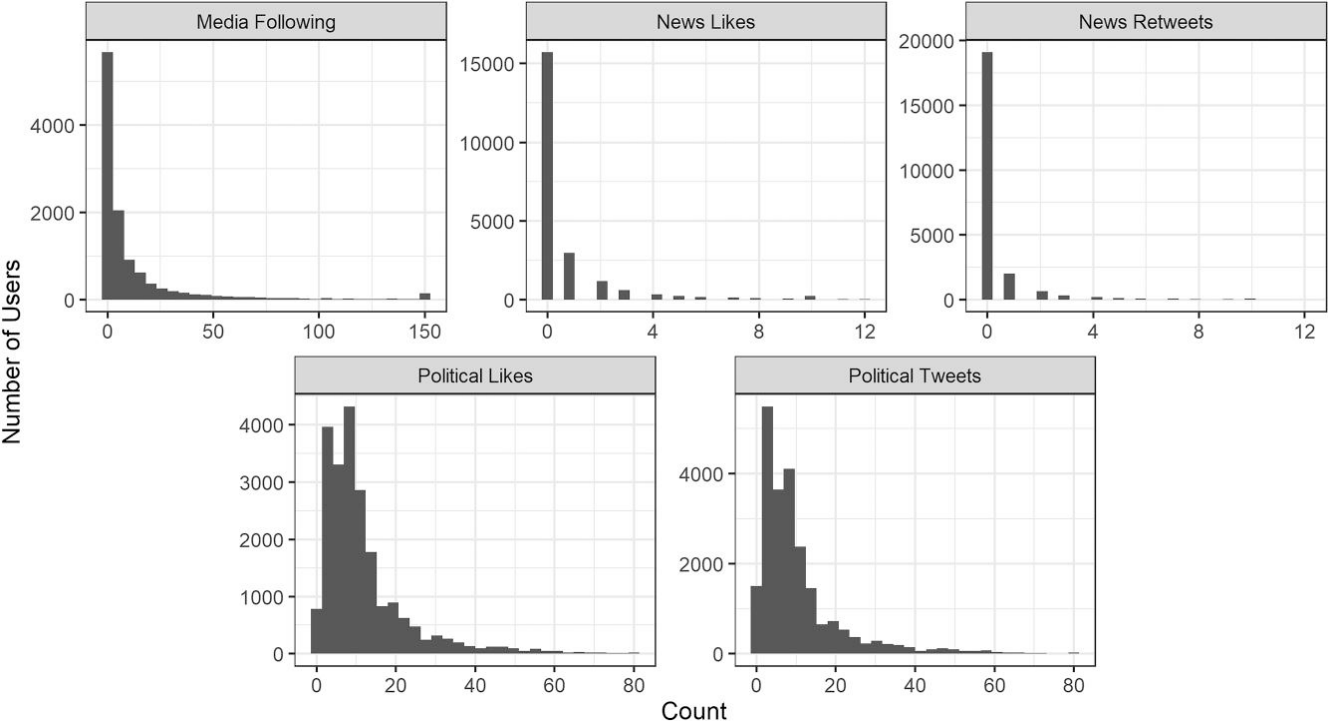
User distribution across pretreatment measures. Followed news media accounts are a count measure based on all recorded accounts followed. News media likes, news media (re)tweets, political likes, and political (re)tweets are measured as a count based on the last 100 likes or (re)tweets made prior to the treatment period.

Looking at the distributions of these variables in Figure 3, we see that many users do not follow any news accounts and do not engage with any content from news media organizations on Twitter. At the same time, the vast majority of users in our sample do like political content and (re)tweet about politics in some form, with most users doing so in between 5 and 20% of their likes and (re)tweets, respectively. At the aggregate level, then, the results suggest relatively limited news engagement, as consistent with prior work (6, 13), and greater engagement with political content.^k^

In terms of user activity during our treatment period, across the two week intervention period, our users (re)tweeted a total of 1,172,143 (re)tweets (396,378 by users in the male bot treatment group, 367,672 by users in the female bot treatment group, 408,093 by users in the control), with 154,878 (13.2%) of those containing text that matched one or more words in our keyword list. Of these matches, 76.67% of (re)tweets were related to the topic of sports, 17.56% related to entertainment and 5.78% to lifestyle. Based on our self-imposed limit of one response per user per 24 h, our bots then responded to 28,211 of these (re)tweets.

### Treatment effects

**Fig. 4.**
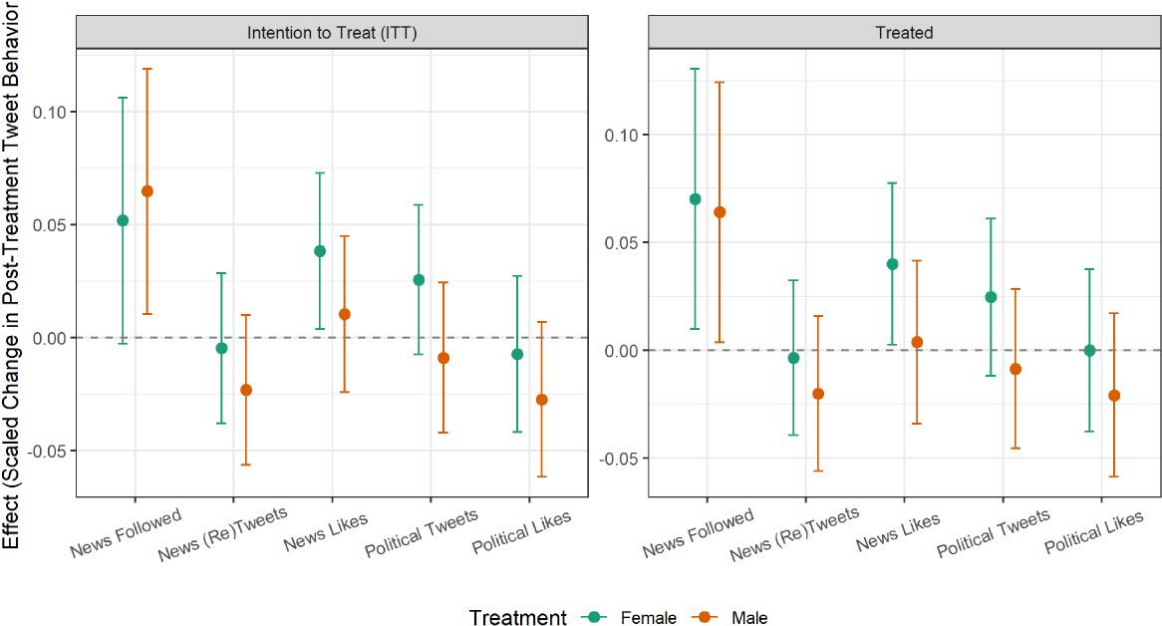
Main effects plot: coefficient estimates and 95% confidence intervals for G-computation after entropy balancing regression models with robust standard errors. Dependent variables taken as the difference between pre- and post-treatment individual user measures. News media accounts followed measured as a count, news media (re)tweets and likes and political (re)tweets and likes measured as percentages.

Modeling the pre- to post-treatment changes in user activity, Figure 4 shows the estimated treatment effects based on a linear regression model measuring the difference in pre- and post-experiment metrics at the user level, with results shown for the male and female treatment groups compared to the control group. The full models are reported in Supplementary Material S8. In the models, the number of news media following is measured as a continuous change in the number of news media accounts followed while news media likes and (re)tweets and political likes and (re)tweets variables are measured as a relative change in the percentage of each measure between pre- and post-treatment.

As our intervention was dependent on users actually tweeting about our keywords during the experimental time period, not all users in the treatment groups received the treatment from one of our bots. To account for this, we estimate two distinct treatment effects: “Intention to treat (ITT)” (i.e. all users from our original randomized treatment groups) and “Treated” (i.e. users in the male and female treatment groups who actually received one or more responses from our bots). Because certain types of users may have been more likely to tweet matching keywords and view the bot responses, simply dropping untreated units would result in an imbalance between the control group and the refined treatment groups, we use an entropy balancing approach (79) to reweigh our “treated” treatment groups relative to the control group. ^l^ The entropy balancing is based on four account level metrics that capture the overall size and activity levels of an account and that are also correlated with the likelihood of receiving and seeing a treatment (total likes, total tweets, total followers, and total followed accounts). For comparability, estimates are standardized with effects interpreted in standard deviation changes.

Figure 4 shows that, in our ITT models, users in the female treatment group liked significantly more content from news outlets compared to the control group, and that those in the male bot treatment group followed significantly more news organizations. The other variables showed no statistically significant change in the ITT models. When examining the users who were indeed treated with bot responses during the experimental period (see the “Treated” models in Figure 4), we see that users in both the female and male bot treatment groups were significantly more likely to follow news media accounts (P=0.05) than those in the control. Each user in the female bot treatment group followed, on average, 0.75 more news accounts than the control, and those in the male bot treatment followed 0.69 more news accounts during the treatment period.^m^ In addition, those in the female bot treatment group liked significantly more news content (with a consistent coefficient of a 0.04 standard deviation increase). Given that the median number of pre-treatment following and liking of news outlets was 0, these effects are meaningful. Nevertheless, although meaningful and significant, these effects were substantively very small. Also, we find no statistically significant effects of our intervention on the three remaining outcomes: (re)tweeting tweets from news accounts, (re)tweeting political content, and liking political content.

In general, then, among those users who actually received our treatments, users followed slightly more news accounts and those who were treated by a female bot additionally liked more news content, suggesting small differential effects based on the gender of the bot.

**Fig. 5.**
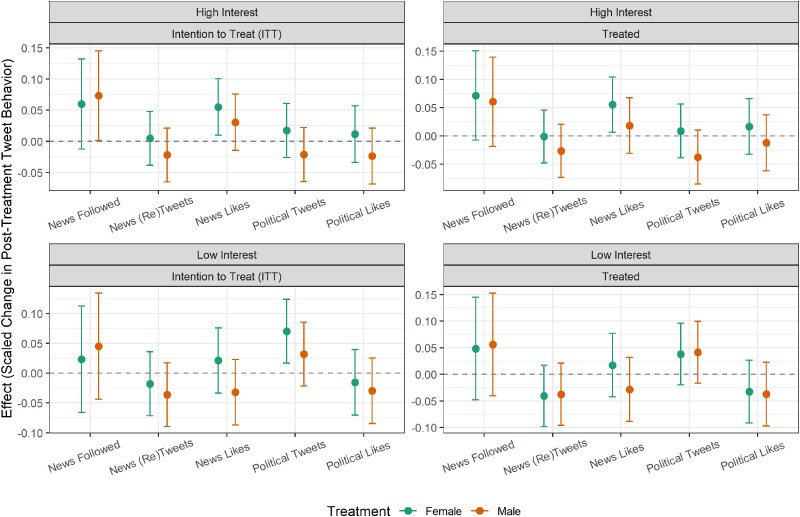
Main treatment effects divided by users’ prior political engagement levels: coefficient estimates and 95% confidence intervals for G-computation after entropy balancing regression models with robust standard errors. Dependent variables taken as the difference between pre- and post-treatment individual user measures. News media accounts followed measured as a count, news media (re)tweets and likes and political (re)tweets and likes measured as percentages.

Given that the average engagement with news and political content was relatively low, the question arises as to which types of users may have been affected by our treatments. Specifically, were the detected increases concentrated among those who were politically engaged already or were the treatments able to trigger some baseline engagement among users who were previously not interested in news and politics? We examine the heterogeneity of the effects by users’ prior on-platform engagement with political content (a binary indicator of whether a user (re)tweeted 5 or fewer vs. more times about politics in the pre-treatment period). Figure 5 shows that the results are only significant in the high political interest group. For those users, the responses from female bots significantly—but again only slightly—increased the liking of news content. The following of media accounts also increased (although due to the reduced sub-sample size, the *P*-value falls to 0.11, despite the estimated standardized effect size actually increasing relative to the overall model). In the low interest sub-group, no effects in the treated model estimations are significant or approaching significance. We also explored whether the treatment effects differed by topic category by running separate models for users who—during the treatment period—tweeted about entertainment vs. lifestyle vs. sports. The results are shown in Supplementary Material S10.

## Discussion

The American public is largely disinterested in politics (29) and the aggregate consumption of news and political information is limited offline (18, 35–37), online (80), and on social media platforms (13, 38, 45, 81). And yet, because news consumption has many beneficial effects—ranging from increased political knowledge and participation, to more stable political attitudes, greater political tolerance, and higher support for democratic norms (19, 25, 82)—the question as to how to incentivize social media users to consume more quality news is important. Because most citizens go online for entertainment (42), this project proposed to link users’ online habits and interests with nudges that could encourage them to consume more news and public affairs information on Twitter.

Our experiment ran for 2 weeks on a sample of 28,457 US-based Twitter users interested in sports, entertainment, and lifestyle. To engage those users with news and politics, we deployed 28 male and female bots trained to contextually respond to the users in online conversations and to suggest topic-relevant sub-pages of verified and ideologically balanced news outlets, as determined by external metrics. As suggested by the literature on “soft news,” this intervention should spark the users’ attention through the connection with their non-political interests and encourage the users to follow and engage with these sources on Twitter. Ultimately, the intervention aimed to put more public affairs information in the users’ feeds, signal to the platform algorithms that the users are interested in news and politics, and eventually lead the users to consume more “hard news.”

Our project offers three key findings. First, our bot-based intervention, and in particular female bots responding to Twitter users with the encouragement to follow verified and ideologically balanced news accounts and to visit topic-specific sub-pages of news organizations led to minimal changes in news and political engagement on Twitter. This suggests that mere bot-based intervention is an insufficient encouragement to change individual media engagement behavior in any pronounced way. As people tend to be resistant to change their habits and as media consumption is a habitual behavior, the finding that our intervention did not increase such aspects of news and political engagement as (re)tweeting news content, (re)tweeting political content, or liking political tweets may not come as a surprise.

That said, our treatments did slightly increase some aspects of news engagement among the treated users. Those users did follow more news accounts (which was the explicit purpose of the treatment) and those who were contacted by female bots also liked more posts from news outlets on Twitter. These effects were small in magnitude, producing a less than 0.1 standard deviation change. Although small, these effects *could* be meaningful in ways that cannot be quantified in this project. In particular, the additional following of news accounts puts more content from these accounts in users’ social media feed. Exposure to such content could over time increase political knowledge and efficacy, generate engagement with the content seen, and serve as a gateway to following additional accounts and consuming public affairs elsewhere. In addition, this increased following and the liking of news content could send the signal to the algorithm that the user is—at least to some extent—interested in public affairs, thereby promoting future recommendations to relevant content and suggestions to follow news or political accounts (83). These subtle and potentially cumulative effects cannot be tested here, and so we invite future work to examine them using longer designs that capture additional variables, both behavioral and self-reported. We also encourage researchers to explore how bots could be designed to incentivize people to follow verified news accounts and click on relevant links more effectively.

Second, the bots presented as females led to more consistent and stronger increases in news following and liking. Despite the fact that politics is still seen as a male domain (57, 58) and despite the evidence that women are more likely than men to experience harassment or hate speech on social media (63–65), our sample was slightly more responsive to the intervention when it came from a female. Because Twitter users are predominantly male (61.2% vs. 38.8%) (84) and because our sample was likely to have even more male users (given that sports was the dominant category), it is possible that men are more open to female bots nudging them and could feel potentially threatened by male bots telling them to follow news. Again, because these effects were small and not very robust, and because this evidence goes against the prevalent finding that politics and the online sphere are spaces where female opinion is disregarded, we encourage more work exploring these potentially differential reactions to pro-social online interventions coming from females vs. males.

Third, the small detected effects were largely confined to the group of users who were already interested in politics. It is those who had previously tweeted about politics who showed the most substantial increases in the liking of news contents as a result of our intervention (among those who were treated). In contrast, the bots failed to encourage users who were not interested in politics, as determined by their previous posting patterns, to follow more news accounts, (re)tweet politics, or like news and political content. This finding speaks to the reinforcement hypothesis (33, 34) and suggests that people who are already politically interested are the ones who become yet more engaged as a result of interventions similar to ours. Although incidental exposure to news and public affairs on platforms—which the comments from our bots effectively created—could serve an equalizing function and pull the previously disinterested citizens back into news and politics (50)—our study adds to the more pessimistic evidence that individuals with high political interest are becoming information richer and more participatory, whereas the ones with low political interest remain politically disengaged (33).

Naturally, the experiment is not free from limitations that offer important directions for future research. It is possible that stronger effects would emerge if we used GPT-4 or other Large Language Models more powerful than our fine-tuned GPT-2. GPT-4 generated replies to users would have likely been even more human-like and better aligned with the original users’ tweets, thus potentially generating stronger effects. In addition, although the experiment ran for 2 weeks, a time frame that is rather extensive, more rounds of user–bot interactions over an even longer time period could have led to more pronounced effects. Because many of the users tweeted relatively infrequently (6,477 users out of 28,457 did not tweet even once during the experiment period) and many (3,674 users out of the remaining 21,980) did not tweet our keywords at all during the treatment period, the treatment may have been too weak to generate effects. That said, the bot responses were capped at one per day to avoid spamming or angering the users. Future studies should examine what “dose” of various social media interventions is most effective.

In addition, it is not certain whether similar effects would emerge on a different platform, in a different time, or in a different sociopolitical context. Twitter is known to be an important channel for political information (85, 86), a key platform for politicians, journalists, and pundits (87), and one where many users express their political opinions (88, 89). As such, engaging users with news may have been more “natural” on and better integrated with Twitter than with Facebook or Instagram, where many users have more closely knit networks of friends and family. We encourage scholars to replicate our results on other platforms. Given the changes to the Twitter API after Elon Musk acquired Twitter, field experiments such as ours and others (31, 90, 91) might be no longer possible.

Lastly, our core focus was on encouraging users to follow news accounts and engage with news and political information on social media, and so we cannot ascertain whether the treatment or the slightly increased news following and liking had any effects on users’ political attitudes. Growing evidence suggest that although various (algorithmic) interventions can powerfully alter users’ on-platform exposures and behaviors, this has no corresponding effects on affective polarization, misperceptions, policy positions, among other survey outcomes (45, 83, 92–94). As aforementioned, however, our treatment could have triggered or enhanced political interest among some users, served as a gateway to hard news, or made users feel politically efficacious, outcomes that we did not measure and that are often overlooked in similar work.

Despite their constrained nature and limited size, the detected effects have implications for research, platforms, and democracy more broadly. As most users do not see or engage with public affairs information on platforms (6, 13) in part because they do not have such information in their social media inventory (14), scholars should explore ways to encourage citizen engagement with news and politics and design (algorithmic) interventions that make such content easily available to users. Although social media researchers disproportionately focus on the (hot and sexy) misinformation and “echo chambers,” these digital problems are relevant to a much smaller subset of the population than the low levels of consumption of verified political information. As such, a shift in focus is needed.

In addition, the fact that enhancing the accessibility of verified and balanced news at least slightly encourages some aspects of citizen news engagement suggests that platforms could (and should) introduce such pro-social interventions toward increasing citizen awareness of public affairs. Naturally, platforms prioritize user engagement over the quality or veracity of information (95). Yet—as other research shows (83)—algorithmic nudges that increase recommendations and exposure to verified and ideologically balanced news do not decrease user on-platform engagement and users report wanting more informative, educational, and verified content on platforms (96). Naturally, more research is needed on how to minimize exposures to harmful content and enhance user engagement with verified public interest information on social media. As most people post about such non-political issues as yoga, baseball, or a recent blockbuster, connecting those interests to public affairs so that to make news and politics more relevant holds some promise.

This and other similar interventions that put at least some public affairs information in the users’ online ecosystem have the potential to minimize polarization and political hostility. Given that a growing group of Americans withdraw from news and politics (29), pulling them back into the democratic process would include more moderate voices in the political arena, minimize the disproportional influence of the more polarized and vocal strong partisans, and make the electoral process more equitable. Given the widespread use of platforms and the various challenges faced by the United States and other democracies, such research is timely and needed.

## Materials and methods

Our experiment was fielded in 2023 January 1 to 2023 February 3 with data collection and processing occurring in January–February 2023. Collectively, across this period, there were five stages for the setup and execution of our experiment. Firstly, we identified keywords across three distinct popular non-hard news topic areas, we then collected our user sample for the experiment, followed by their pre-treatment Twitter information, we then ran our news bot intervention on their relevant tweets, and, finally, we collected their post-treatment data. We leverage Tweepy (97) and several Twitter v1.1 API tokens to perform all experimentation. Details on which API calls we used to conduct the experiment can be found in Supplementary Material S4D. We expand on the process below.

We identified US-based Twitter users who actively tweeted about one of three topics: sports, entertainment, and lifestyle, across a one week period in September 2022. To do this, we created a list of 1,763 keywords generated using word embeddings and manual additions (e.g. current movies and television series, athletes, brands; see Supplementary Material S1A for details; keywords broken down by topic are available at Github). We collected our initial user base by scraping the user IDs of all Twitter users who tweeted our keywords at least once in a 7 day period (using API.search_tweets()), with location and language filters to ensure that only users based in the United States and tweeting in English were included (N=118,032). We used the package geostring (98) and sPaCy’s Language Detector (99) to filter location and language, respectively. We then excluded those who tweeted only once during the 7 day period, as these infrequent users were relatively unlikely to be active during the treatment period. To minimize the chances that power users or administrative accounts (e.g. celebrities, brands, or organizations) are disproportionately represented in our sample, we also excluded users who tweeted more than 20 times (Nremaining=63,843) and those who were in the top 10th and 90th percentiles of followers and followees (i.e. those who had fewer than 79 or more than 16,500 followers and those who followed fewer than 127 or more than 4,500 accounts). Finally, we removed all users with a botometer score (72) of more than 0.60 to minimize the inclusion of bots. This resulted in a final sample of 28,457 active nonbot US users known to tweet about the three topics more than once a week. More details available in Supplementary Material S1B.

Having identified our user pool, we then assigned these users to one of three treatments (a male bot, a female bot, and a control group). Randomization was successful on a range of account level metrics (the total number of followed accounts, total number of followers, total posts, and total likes) as well as central pre-treatment metrics (the number of news media accounts followed, the number of recent likes of news media posts, the number of (re)tweets of posts from media accounts), ensuring balance across groups in terms of existing engagement with news media content. To account for the volume of messaging required, we created multiple bot accounts per treatment group (14 male and 14 female). These bots were designed to be realistic at a visual level, with each bot having a clearly gender definable headshot picture and a clearly gender identifiable name. In order to better comply with Twitter’s Terms of Service, we included the following in the bio of the accounts “This account is designed to share verified, factual, and quality news. It is operated by researchers @ University of California, Davis.” See Supplementary Material S1D for more details which websites we used to create the accounts.

To generate responses to the treated users, we leveraged GPT-2 models (73). This model was fine-tuned on Reddit comments by Microsoft and was designed to be conversational in nature. Before sending the Tweets to the GPT-2 model, we removed all URLs and special characters and discarded the GPT-2 response if it contained language pertaining to Reddit (such as upvote, subreddit, etc.), kept on repeating the same text, or used profanity. In cases where responses were discarded, the contextual text was replaced by a randomly selected hardcoded template response. In addition to the GPT-2 based reply to each user’s tweet, we hardcoded two elements into the response. We encouraged users to follow a news media organization (e.g. “follow @wsj” or “follow @nyt”) and to visit a link to a relevant sub-section of a verified and ideologically balanced news source (e.g. an entertainment/sports/lifestyle section of the Wall Street Journal or the New York Times). More details on this process can be found in Supplementary Material S2. As mentioned in the main text, the bot responses were encouraging users to follow and visit news media organizations that have been established to be verified and ideologically balanced. All the sources, their reliability and bias scores, and the URLs to the relevant sub-sections are presented in Supplementary Material S3A.

Every 8 h, we scraped the timelines of all users using API.user_timeline(). Tweets matching one of our topic keywords would then receive an automated reply from an assigned bot account, which contextually and dynamically matched the reply to the original tweet of a user using API.update_status(). Each response also encouraged the user to stay up to date with the news and visit a link to a topic-relevant sub-section of a news source from our list, as aforementioned. We limited the number of responses to one per day, so as to ensure that the users who tweet using our topic keywords multiple times a day would not be irritated or seeing our responses as spam. The scraping and response cycle ran continuously for two weeks. After this time period, the treatment to all groups was terminated.

### Outcome measurement

We measure three different variables across conditions pre- and post-intervention, namely (i) how much (a) political content (b) content from news organizations users (re)tweet (i.e. tweet, retweet and quote tweet), (ii) how much (a) political content (b) content by news organizations they like, (iii) how many news accounts they follow. To do so, we collected the following for all subjects *before* the intervention: (i) their last 100 (re)tweets before the start of the experiment, which we classify as (a) political or not with a BERT classifier; and which we (b) categorize as coming from a news/political/media account or not (based on an extensive list of 5,400 US news organizations and 5,341 Twitter handles, as well as a list of political/media personalities) Github (ii) the last 100 “likes,” which we classify as being on content that is (a) political or not with a BERT classifier; and on content which we (b) categorize as coming from a news/political/media account or not (based on the same list); (iii) the list of accounts they followed at the start of the experiment, which we use to determine the number of news/political/media accounts followed (based on the same list). *After* the experimental manipulation, we collected equivalent variables.

The API call we used to get the followed accounts was API.get_friends_ids(). The call for likes was API.get_favorites() and the call for (re-)tweets was API.user_timeline(). The final counts collected were as follows: followed accounts (pre N=6,536,692, post N=17,286,211), (re-)tweets (pre N=2,285,401, post N=2,201,009), and likes (pre N=2,927,951, post N2,846,354). More details can be found in Supplementary Material S4.

This project was approved by the Ethical Review Board of the University of Amsterdam, Amsterdam School of Communication Research (ERB number 2022-PCJ-15366). The study was determined to qualify as standard research and informed consent was not required. Given that the number of participants in this study was initially estimated at 40,000, it was practically not possible to collect consent form everyone whose data would be used. Also, sending informed consent via direct messages on Twitter would violate the terms of service of Twitter and potentially result in our accounts being immediately banned. Even though consent could not be collected, users could opt-out of the study by sending us a direct message on Twitter. In addition, it was deemed in the public interest to conduct the research and the gains from the study far outweighed whatever risks and any potential harms to participants, which would not be larger than what individuals experience in their ordinary life. In addition, the bot accounts explicitly identified as bots that were created by researchers.

## Supplementary Material

pgae368_Supplementary_Data

## Data Availability

The analytical code, classifiers, and anonymized data are made publicly available at Code and Data.

## Notes

a This is the case online more broadly: only between 2% (16) and 7–9% (17) of all URLs visited by large samples are news, and—across mobile and desktop—news comprises only 4% of total consumption (18). Although news consumption is greater on television than online, it is still overshadowed by entertainment and other content categories (18).

b We recognize that heavy news consumers tend to be more partisan, more polarized, and more hostile toward the other side (29). We are not targeting those heavy news consumers, instead putting some public affairs information in the online ecosystem of those who most frequently engage with nonpolitical topics on social media. This rather “minimal dose” of news is unlikely to lead to polarization or hostility, as we also note in the Discussion.

c We use the old company name, given that the platform was named Twitter during data collection. We focus on Twitter because it is an important channel citizens use to get news and political information (30) and because it made the field experiment possible at the time of the study (31).

d Our intervention is rooted in the idea of nudging, or altering the choice architecture to influence individual behaviors (32). We acknowledge some criticisms of nudging, which is built on libertarian paternalism and presumes top-down decisions about what is good for an individual. We also note that in our approach, the users can choose not to react, we are not restricting users’ freedom or choice architecture, and we are not excluding any choices for the users.

e We acknowledge that some people *choose* to *actively* avoid news because it is negative and generates stress and anxiety (37). This active news avoidance typically occurs amongst those who (used to) frequently consume news and are taking a break. Many people, however, are passive news avoiders, who prefer nonpolitical topics and/or may not see news as personally relevant. Those people do not have the opportunities to encounter news on platforms (38, 46, 47). Our intervention targets this latter group and is unlikely to negatively influence their well-being as it does not entail a heavy stream of news.

f For consistency and to not introduce confounders, all profile pictures of the bots were white.

g At the time of the study, higher quality open source models (like Llama2) were not available and it was not feasible to use GPT-3 due to the scale of our experiment and the associated costs.

h A recent analysis found high levels of agreement between news domain quality ratings by six organizations, Ad Fontes included (75). Because Ad Fontes ranks both reliability and bias, which were of interest to this study, we used their ratings.

i Followed Account collection using the Twitter API was inconsistent over time, preventing us from collecting all the followed accounts for the entire sample. Specifically, Twitter API’s get_friends() endpoint was returning inconsistent results during the pretreatment stage. We validated the results several times after the experiment, but during the pretreatment collection, the API call returned only a subsection of a user’s following list. As such, we have full followed accounts data for 11,254 of our users collected at both the pre- and post-experiment stages, and we therefore base results for this measure on only these users. Within these collected users, some exhibited unusually high or low decreases in total followers, suggesting either unusual account activity or unresolved inconsistency in API collection results. We therefore only include those whose total followed accounts changed by less than a 50% increase and more than a 20% decrease (approximately the 10th and 90th percentiles) and increased by an absolute count of less than 200. We also show robustness checks for alternative cutoffs around these follower numbers in Supplementary Material S9.

j This low percentage may be a feature of our design and sampling strategy, such that we purposefully selected users based on their frequent posting about sports, entertainment, and lifestyle.

k We note that these relatively high percentages may be due to the false positives in our estimates, see Supplementary Material S4C, which—however—are unlikely to have any effects on the treatment effects.

l For modeling consistency, we use entropy balancing across all models. Because the ITT group was successfully randomized prior to the study, the effect of balancing here is exactly 0, with all units in the ITT group having a weight that is either precisely or rounds to 1. Therefore, in practical terms, entropy balancing is only applied in the case of the “Treated” models.

m We also test whether users followed the specific news accounts in our pool of suggested accounts. Here, we find a directionally positive increase for both the male and female treatment groups compared to the control group, although the *P*-values in the treated models not statistically significant (0.139 and 0.181). We show these full results in Supplementary Material S9.

## Preprint

This article was posted on a preprint: https://arxiv.org/abs/2403.13362 DOI: arXiv:2403.13362.
