# Supplementary material for: Incentivizing news consumption on social media platforms using large language models and realistic bot accounts
Source: PNAS Nexus. 2024 Aug 23;3(9):pgae368. doi: 10.1093/pnasnexus/pgae368 (PMC11404517; doi:10.1093/pnasnexus/pgae368)
Supplement: pgae368_Supplementary_Data [file pgae368_supplementary_data.pdf]

Supplementary Information for

Incentivizing News Consumption on Social Media Platforms Using Large Language Models and Realistic Bot Accounts

Hadi Askari, Anshuman Chhabra, Bernhard Clemm von Hohenberg, Michael Heseltine, Magdalena Wojcieszak

To whom correspondence should be addressed, Magdalena Wojcieszak. E-mail: mwojcieszak@ucdavis.edu

This PDF file includes:

Supplementary text
Figs. S1 to S2
Tables S1 to S15
SI References

Supporting Information Text

1. Fielding the Experiment

There are five distinct stages of the experiment. First, we identify keywords across three distinct topic areas. Second, using these keywords, we collect the user sample for the experiment and then collect the relevant pre-treatment on-platform behavioral metrics of the users. Then, we run the intervention on their relevant tweets, and finally collect the users' post-treatment data. Each of these phases will be outlined in detail below:

A. Identifying Keywords. First, we compiled three lists of relevant keywords, one each for the categories of "lifestyle", "entertainment" and "sports". These keyword lists, containing 200, 820, and 743 keywords, respectively, were compiled using both word embeddings and manual additions by the authors such as the latest most relevant shows, movies and athletes etc. We include only keywords predominantly relevant to the United States audience (e.g., only sports stars based in the U.S., or shows trending in the U.S.). A full list of keywords can be found here Github.

B. Selecting Users. Examination of the Twitter user-base in the United States suggest that a majority of users tweet infrequently and exhibit only moderate level of engagement with content from other users, while a highly active minority of users generate the vast majority of content on Twitter (1). Therefore, in selecting our sample of users, two main criteria were considered. First, it was important to identify *active* users who were likely to post topic-relevant content during the time period of the experiment and could therefore be treated (i.e., the bots' responses were only triggered if a user tweeted one of the keywords on our list). Second, it was also important to limit the number of high-volume power users or users who generally posted with excessive frequency, as these users were likely to be brand representatives, communication accounts of sports teams, etc.

To do this we ran a scrape of all tweets which mentioned one or more of our keywords beginning one week before our treatment interventions commenced. To ensure that we collected only the most relevant users, we firstly refined our search to include only tweets from users whose accounts were geo-located in the United States by performing fuzzy dictionary matching of the users' location to known geographic locations (2). We also excluded tweets which were not primarily written in the English language using sPaCy's Language Detector (3). We excluded verified users and also excluded any username that included the word bot. Additionally, we wanted users that were more likely to post their own tweets rather than simply reply to others so we collected users from only the Tweets that were not replies to other tweets. After collecting these tweets for one week, we were left with tweets from a total of 118,032 unique users.

Having identified these users, we then narrowed our sample to contain only users that were suitable for targeting in our experiment. Firstly, users who only tweeted once about any of our keywords during the time period were removed - given their low frequency of relevant tweeting, these users had a relatively low likelihood of tweeting about our keywords during the experiment period and therefore being exposed to our treatment. On the other end, users who tweeted too much were also removed. To do this, we kept all users up to and including the 90th percentile of most frequent tweeters. This meant that all users who tweeted about our keywords 10 times or less during the collection week remained in our sample. The removal of those above this threshold helped significantly lower the number of spam users and bots contained within our sample.

After trimming the users based on tweet frequency, we minimized the number of bot accounts remaining in our sample. From our remaining users, we ran the accounts through Botometer (4), a tool which checks the activity of Twitter accounts and gives them a score based on how likely they are to be bots. Based on the resulting Botometer score, all accounts with a score of 0.6 (out of 1) or higher were removed from the sample.

Having trimmed the users for location, tweet frequency and bot likelihood, we were left with a final sample of 28,457 users. These users were then randomly assigned to one of the three groups: two treatment groups, each one receiving automated responses from bots presented as female or male, and one control group, which did not receive any interventions during the experiment.

C. Establishing Baseline User Behavior. We collected user-level metrics to establish broad baseline news engagement information about our final user pool. The following information about each user was collected prior to the commencement of the experiment: (1) how many news accounts they follow, how much (a) content from news organizations and (b) political content users (re)tweet (i.e., tweet, retweet and quote tweet), (2) how much (a) content by news organizations and (b) political content they like. To do so, we collected the following for all subjects: (1) the list of accounts they followed at the start of the experiment, which we use to determine the number of news/political/media accounts followed, (2) their last hundred (re)tweets before the start of the experiment, which we (a) categorize as coming from a news/political/media account or not (based on an extensive list of US news organizations and their Twitter handles Github and which we classify as (b) political or not with a BERT classifier (5); (3) the last hundred "likes", which we classify as being on content which we (a) categorize as coming from a news/political/media account or not (based on the same list) and on content that is (a) political or not with a BERT classifier (5).

D. Bot Creation. : We created email accounts using mail.com (6) and generated phone numbers for verification using textverified.com (7). We decided to keep the name and image of our bots as Caucasian/white to not introduce additional confounders. We generated images for our bots using this-person-does-not-exist.com (8). In order to better comply with Twitter's Terms of Service, we included the following in the bio of the accounts "This account is designed to share verified, factual, and quality news. It is operated by researchers @ University of California, Davis". We created 28 different bots, splitting them between male and female.

E. Experimental Design. For the experiment, we scraped the timelines of all users in our sample on a regular basis (every 8 hours). If a tweet from a given user contained one of our keywords (defined based on exact matching criteria to avoid false positives), then, based on the keyword topic and the bot group the user was assigned to, our system automatically generated a response to their tweet. This response contained an automatically generated comment, tailored to the original tweet (more detail below) and a link to one randomly selected news outlet from our list of media outlets (See Table S2 below), with the link specifically directing to the sport, entertainment, or lifestyle section of that outlet, depending on the keyword group mentioned in the original tweet.

For the news links contained within our responses, we compiled a list of ostensibly ideologically centrist mainstream news outlets that also contain soft news or non-political news sections on their websites. This final list comprises of 24 outlets, with the full list viewable in table S2. In randomly sampling from an extensive range of outlets, we avoid identifying only particular effects relating to specific news outlets, with which individuals may be more or less prone to engage or avoid based on their prior beliefs about the outlet.

To avoid the perception of spam, we generated a maximum of one reply for each user in a 24-hour period - additional tweets from a given user in this 24-hour window which also featured one of our keywords were still collected in our database, but did not receive a response of any kind. To allow sufficient time for the experiment, both in terms of number of treatment interventions and for behavioral responses, the scraping and response cycle ran continuously for a period of two weeks. After this time period, treatment to all groups ceased and post-treatment data were collected.

F. Collecting Post-Treatment Data. After the experimental manipulation, we collected the same information as in the pre-treatment period, upon the completion of our experiment. This included the accounts followed list of the users the and their last 100 likes and tweets (i.e., tweet, retweet and quote tweet). It should be noted that Twitter's API (get_friends()) returned inconsistent accounts followed for all of the users per request so we had to run multiple iterations to collect the accounts followed of 11,254 users in our sample.

Collectively, this pre- and post-treatment information allowed us to ascertain how user behavior changed after our treatment period with regards to following news organisations and tweeting about news, as well as data on the extent to which individuals actually engaged with our messages.

2. Generating Realistic Bot Responses

As our central treatments depended on users engaging with Twitter messages from our bots, it was important for our generated responses to be realistic in order for the field experiment to be successful. To achieve this we employed the *DialoGPT* (9) model to generate our responses, which is a large-scale pretrained model released by Microsoft.

DialoGPT is a fine-tuned version of OpenAI's GPT-2 (10) and has been trained on 147 million *multi-turn* dialogue scraped from Reddit discussion threads. In particular, DialoGPT is especially useful for *multi-turn* conversations as it can retain a set of historical outputs and generate a response based on these. However, we found that DialoGPT was highly successful at generating realistic responses for our case as well, where we had a single-turn conversation as the bot responds to a pre-selected user's tweet. Note that we also cleaned the input tweets to remove special characters, URLs, and before they were provided to the model as input.

Furthermore, in an automated fashion, we checked the output generated by the model to ensure that it met some minimum requirements:

- DialoGPT can at times generate responses that mimic the input, or fallback to some consistent generic response (such as *"I am not sure if you're serious or not, but I'm going with the latter"*). Such responses could come across as spam-like especially if they were to be repeated often.

- We also checked if responses contained profanity. We employed the widely used and open-source `profanity-check`[*] Python library for this purpose.

- We further checked if responses contained terminology commonly associated with Reddit (such as 'upvoting', 'downvoting', 'subreddit', etc). Such responses are an artifact of the training data of the model which originates from Reddit, but did not make sense if used on Twitter.

Therefore, in case the model generated one of the above responses, we fell back on simple template responses that we manually created prior to the start of the experiment. In case of failure, we randomly sampled from these template responses Github, thus keeping the bot outputs new and different when they engaged with the users in the field. Moreover, employing such a *hybrid* approach was advantageous, because it allowed us to avoid known issues of inconsistency, fabrication, and repetition that generative language models (11) (such as GPT-2) are prone to.

Collectively, this provided us with a system of auto-generating highly relevant replies to each users tweets, with content that was tailored to the tone and content of their message, while avoiding irrelevant or spam-like syntax. An example of this is shown in table S7 below:

We also tried a number of other models as an alternative to DialoGPT. We first fine-tuned vanilla GPT-2 on a large collection of tweets but found that this model generated noisy outputs which were quite inconsistent, and mostly informal. We

[*] https://github.com/vzhou842/profanity-check

Original Tweet	Bot's Reply
Anybody who says college basketball is better than the NBA needs to be put away.	I think college basketball is better. To learn more about sports click https://www.nytimes.com/section/sports and follow @nytimes.
I mean, the USA pretty much dominates Olympics men's basketball and there's no push to remove that event.	I think it would be deeply unpopular with the NBA players. To learn more about sports click https://www.theguardian.com/us/sport and follow @guardian.

Table S1. Illustrative examples of user Tweets with relevant responses

also came up with simple templates that the model could fill-in with a satisfactory response based on the input tweet. For this, we fine-tuned a popular infilling language model (12), but found that most generated responses were quite poor and not good enough for our use case. Finally we tried an existing pre-trained GPT-2 model (13) fine-tuned on a dataset which consists of 143,000 news articles from 15 USA publishers between 2016-2017. This also did not perform satisfactorily in our validations of the outputs on some sample tweets. In comparison to these approaches, DialoGPT had more consistent and higher quality generated responses.

3. Validating the bot responses

To validate the responses, we sampled 500 random tweets and generated responses for them using our hybrid approach. Each of the authors then individually went through all the responses and annotated each response as either 'satisfactory' or 'unsatisfactory'. We then take a majority vote for each sample to find out the total number of 'satisfactory' and 'unsatisfactory' replies. A majority vote of 'satisfactory'/'unsatisfactory' for a sample here constitutes ≥ 3 annotations of 'satisfactory'/'unsatisfactory.' We found that the approach generated 407 'satisfactory' responses and 93 'unsatisfactory' responses resulting in an accuracy of $407/500 = 81.4\%$ for the approach at generating 'satisfactory' responses.

A. News Organization Selection. To ascertain that the users received tweets promoting quality and verified news media organizations, we selected news media outlets using Ad Fontes' Media Bias Chart (14)[†]. Ad Fontes scores were based on manual labeling of articles, radio, TV, and videos for numerous news sources. For each, Ad Fontes selects a sample of articles that are most prominently featured on that source's website over several news cycles (min. 15 articles/source, several dozen articles for top 100 sites/each, and over 100 articles for the largest sources, such as the New York Times and Washington Post). Ad Fontes uses a multi-person rating per article system to minimize the impact of any one person's political bias on the published rating. Each individual article and episode is rated by at least three human analysts from across the political spectrum (balanced right, left, and center, based on self-reports). The scores are assigned along two dimensions: reliability (from "contains inaccurate/fabricated information" to "original fact reporting") and political bias (from "most extreme left" to "most extreme right"). We selected reliable and generally ideologically balanced sources, with a reliability score higher than 40 and a bias score between -18 and 18. For these sources, we also identified their corresponding Twitter handles and recommend news links of their "sports", "lifestyle" and "entertainment" pages. Table S2 shows the details.

Media Outlet	Credibility Score	Bias Score	Twitter Handle	Entertainment	Lifestyle	Sports
Vice	41.42	-10.41	@VICE	URL	URL	-
Vox	40.86	-10.23	@voxdotcom	URL	-	-
LA Times	45.16	-10.13	@latimes	URL	URL	URL
Washington Post	43.82	-8.83	@washingtonpost	URL	URL	URL
CNN	42.89	-8.63	@CNN	URL	URL	URL
The Guardian	43.64	-8.54	@guardian	URL	URL	URL
The New York Times	44.72	-7.81	@nytimes	URL	URL	URL
Buzzfeed News	43.22	-7.8	@BuzzFeedNews	URL	-	-
Time Magazine	43.99	-7.06	@TIME	URL	-	URL
NBC News	45.67	-6.78	@NBCNews	-	-	URL
MarketWatch	44.33	-5.08	@MarketWatch	-	URL	-
USA Today	45.72	-5.07	@USATODAY	URL	URL	URL
NPR	46.23	-4.87	@NPR	URL	URL	-
ABC News	46.68	-4.5	@ABC	URL	URL	URL
Al Jazeera	45.57	-4.4	@AJEnglish	-	-	URL
Axios	45.66	-4.35	@axios	URL	-	URL
CBS News	46.87	-3.6	@CBSNews	URL	-	URL
Forbes	43.58	-3.46	@Forbes	URL	URL	-
Insider	43.32	-2.33	@thisisinsider	URL	URL	URL
AP	49.33	-1.86	@AP	URL	URL	URL
Reuters	48.79	-1.31	@Reuters	-	URL	URL

[†] https://adfontesmedia.com/interactive-media-bias-chart/

Wall Street Journal	45.98	4.72	@WSJ	URL	URL	URL
Fox Business	43.38	5.55	@FoxBusiness	URL	URL	URL
Christianity Today	41.92	6.26	@CTmagazine	URL	URL	URL

Table S2. News Media Sources selected in our experiment

4. Outcome Variables

We measure outcomes, across separate models, using five key dependent variables which reflect a given users engagement with news and politics: 1) News Accounts Followed 2) News (Re)tweets 3) News Likes 4) Political (Re)tweets 5) Political Likes. Details of how we collected and classified each of these variables is outlined in detail below.

A. Accounts Followed. First, we measure how many news and media accounts are followed by an individual user. To collect this information, we pulled the full list of accounts followed (referred to as friends in the Twitter API lexicon) by all users in our user pool. We then matched the account IDs for all user accounts followed to an extensive list of media Twitter handles, which were hand compiled by the authors. Overall, we identified 5,341 Twitter handles of all news organizations/associated people on our list. This comprehensive list of news domains is made publicly available on Github. From this, we then construct a count variable for each user reflecting the number of media accounts followed.

B. News Re(tweets) and Likes Identification. To identify whether users followed, tweeted about, re-tweeted, or liked content from news media organizations, we relied on our extensive list of news accounts created and curated for the larger project. The list is made publicly available on Github. We started with a list combining Amazon Alexa's top 1000 most visited domains, the top 1000 most frequently shared domains by politicians on Twitter, and the top 1000 most frequently browsed domains in online behavioral trace data spanning 9 months and from a large U.S. adult sample (15). Multiple coders coded whether a domain was a local, national or international news organization, in which case it was kept on the list. Given the size of the US and its many local news markets, we completed the list with a compilation of local and national newspapers from usnpl.com, and a list of local TV stations and their web domains from officialusa.com. The final list contains a total of 5341 news organizations. In this project, we match all tweets to whether they were a retweet of a user ID from our media list. Similarly, we match all likes to whether the liked tweet was from one of these accounts. As not all users liked and (re)tweeted the same number of (re)tweets in the post-experiment collection period, we convert the raw count of media (re)tweets and likes to a percentage.

C. Political Content Classification. To determine whether participants tweeted about, retweeted, or liked content about political issues, we developed a neural binary classifier. We conceptualize 'politics' rather broadly: tweets considered as political include references to both political figures, policies, elections, news events, and specific political events *as well as* issues such as climate change, immigration, healthcare, gun control, sexual assault, racial, gender, sexual, ethnic, and religious minorities, the regulation of large tech companies, and crimes involving guns.

To train our political model, we fine-tuned the RoBERTa NLP transformer-based model proposed in (16). RoBERTa builds upon BERT (17) and changes key hyperparameters (higher learning rates, mini batches, different pre-training procedure, etc.) for improved performance. To fine-tune, we first collected explicitly political and non-political comment data from Reddit. We collected a total of 9063 comments from the political subreddits: r/Socialism, r/conservatives, r/Conservatives, and from the non-political subreddits: r/soccer, r/nba, r/OnePiece. These were then annotated by trained undergraduate and graduate students and labeled as "0"("1") for "non-political"("political") comments. Manual annotation was required because there could be significant subtleties in deciding political ideology of comments. For fine-tuning the RoBERTa model, we trained for 3 epochs, and employed a learning rate of 0.00002, batch sizes of 16, tokenization max length of 128, and gradient accumulation on each step.

Note that our original goal was to train a model that is highly performant irrespective of text type and social media platform. Therefore, we utilized a combined test set containing 3885 manually annotated comments, with 901 comments collected from YouTube, 594 comments from Facebook, and 2,393 Reddit comments (similarly collected as for the training set). With our trained model, we obtain an accuracy of 91.47%, precision of 90.34%, and 90.77% recall on this platform-wise out-of-domain test set.

We have validated the classifier across social media platforms to ensure that the model performs well across platforms. For Facebook comments, we achieve an accuracy of 86.00%, precision of 86.30%, and recall of 85.90%. For YouTube comments, we achieve an accuracy of 88.67%, precision of 87.67%, and recall of 87.87%. For Reddit comments, we have accuracy of 93.77%, precision of 92.25%, and recall of 92.95%. We additionally manually validated the classifier on a sample of 200 tweets from our data, finding agreement similar to that for the other platforms. In particular, 39 out of the 200 labels by the classifier were mislabeled. These were mostly false positives. For instance, tweets mentioning "breaking" or "breaking news," "defense," "union," or "weed" (because the classifier was trained to categorize issues related to drug legalization or abuse as political), or "taxin" or "dmv" (which can be seen as related to taxes or governmental agencies) were incorrectly labeled as political (see a few examples below). This slight over-estimation of political posts is unlikely to affect the presented results because we are finding null effects on all the outcomes related to posting about or liking political content.

Examples of non-political posts classified as political:

- It's not even just that. It's when someone does need to get by and they act like that person is wrong for it and try to shame them online.
- im good on dmv trap flowers he b taxin get me some sams or costco roses
- Smelling weed in public for me is like smelling the little baked pie on the windowsill in cartoons
- I'm genuinely concerned that without outside interference, she's gonna stay with the guy and "hope he changes".

- I've seen this shit happen before. The abused person gets stuck in this loop because they believe that eventually their abuser will change.

- very sad to report that Shaggy died while trying to retreat from Lyman after his Officers abandoned him

- This picture was taken this evening in the skies over Billings, Montana.

- BREAKING: As reported by SummerSlam is coming to in Detroit, MI on Saturday, August 5.

- has been a staple for our Defense. Last year was on the way to becoming all pro then injured his foot.

We used this final trained model for inference on our Tweets. The contents of our likes and Tweets were fed to the classifier after removing all emojis and URLs and a political classification of either 0 or 1 was obtained. The overall count of political content for each user is then converted to a percentage of (re)tweets and a percentage of likes.

D. Collecting Metrics. We used Tweepy and several Twitter v1.1 API tokens (18) to conduct the experiment and collect the metrics. All tokens were required to have read and write access. In order to maximize efficiency, rotated through them while using I/O multi-threading in Python. The different Tweepy end-points we used are as follows:

- **API.search_Tweets()**: We used this end-point to look for users that Tweet about our keywords while we were collecting users for our study.

- **API.user_timeline()**: We used this end-point to scrape the last Tweets that a user posted. We used the "since_id" parameter to only collect the new tweets that were posted after our previous scrape in the "During experiment" phase. We also used this end point to scrape the last 100 tweets that the user tweeted before and after our experiment to later classify as news and political related or not.

- **API.get_favorites()**: This end-point was used to collect the last 100 likes of a user pre- and post- experiment. The text of those likes was then classified as news or political or not.

- **API.update_status()**: This end-point was used to tweet our treatment at the users from our bot accounts.

- **API.get_friend_ids()**: We used this end point to collect the accounts that our users followed pre- vs post- experiment. We experienced issues with the Twitter API not returning the following lists of all the accounts queried. The post collections had to be repeated for several iterations. We used the "media_user_id" column in our compiled list of U.S. media sources to classify the quantity of media accounts followed pre- vs post- experiment. Github

Metric	Control (Treated)	Female (Treated)	Male (Treated)
**Pre News Accounts Followed**	13.98	12.29	13.31
**Post News Accounts Followed**	22.68	22.75	22.56
**Pre News Likes**	0.82	0.72	0.78
**Post News Likes**	0.85	0.87	0.79
**Pre News (Re)tweets**	0.38	0.33	0.38
**Post News (Re)tweets**	0.22	0.20	0.22
**Pre Political Likes**	11.85	10.67	11.00
**Post Political Likes**	12.23	11.19	11.20
**Pre Political Tweets**	10.48	9.52	9.81
**Post Political Tweets**	9.91	9.22	9.26
**Total User Count**	**9584**	**5354**	**5214**

Table S3. Mean Metrics Pre vs Post Experiment.

E. Sentiment Analysis of Replies. We further evaluated the sentiment of the replies our bots received. We collected a total of 241 (99 Male and 142 Female) responses to our bots Tweets and performed Sentiment analysis using a RoBERTa-base model trained on 124M tweets from January 2018 to December 2021, and fine-tuned for sentiment analysis with the TweetEval benchmark (19, 20). For the Tweets that only had a reply in a GIF or an image, we manually replaced the image/GIF with text representing the sentiment of that image.

Sentiment	Male Bot Replies	Female Bot Replies
Positive	18 (18.18%)	21 (14.78%)
Negative	49 (49.49%)	65 (45.77%)
Neutral	32 (32.32%)	56 (39.44%)
**Totals**	99	142

Table S4. Sentiment Analysis of Responses

5. Account-Level Measure Balance Across Treatments

To verify the balanced randomization of user accounts across treatments, we estimated treatment-level averages (means) of 5 core account metrics, shown in S5. We also tested the pre-treatment balance of our five core user media and political engagement measures. The results show that good balance was achieved across all treatment groups.

Treatment	Listed (Count)	Likes (Count)	Tweets (Count)	Following (Count)	Followers (Count)
Control	25.20	42715.39	44948.89	1076.946	1601.790
Male	23.22	42682.67	44372.48	1071.389	1563.406
Female	24.34	43433.77	45003.21	1074.581	1583.167
ANOVA	0.87	0.368	0.0721	0.108	0.281

Table S5. Pre-Treatment Account-Level Variable Balance by Treatment

Treatment	Following	News (Re)tweets	News Likes	Political Tweets	Political Likes
Control	12.70	0.8244435%	0.3851181%	11.85236%	10.48780%
Male	12.90	0.8080164%	0.4061403%	11.93191%	10.53004%
Female	12.73	0.7903379%	0.3918830%	11.81152%	10.42875%
ANOVA	0.922	0.716	0.684	0.857	0.811

Table S6. Pre-Treatment Activity-Level Variable Balance by Treatment

6. Pre- and Post-Experiment Comparison of User Behavior

Table S7. Mean Metrics for the Pre- and Post- Experiments.

Timing	Metric	Control	Female	Male
**Pre-Experiment**	Followed Accounts	13.98	13.71	13.69
	News Likes	0.820%	0.790%	0.810%
	News (Re)tweets	0.380%	0.390%	0.410%
	Political Likes	11.85%	11.81%	11.93%
	Political Tweets	10.48%	10.43%	10.53%
**Post-Experiment**	Followed Accounts	22.68	22.30	22.11
	News Likes	0.850%	0.930%	0.840%
	News (Re)tweets	0.220%	0.210%	0.230%
	Political Likes	12.23%	12.18%	12.05%
	Political Tweets	9.910%	10.15%	10.02%

On average, our users followed 13.71 (Female Treatment group) and 13.69 (Male Treatment group) news accounts prior to our treatment. This number increased to 22.30 (Female Treatment Group) and 22.11 (Male Treatment Group) after the conclusion of our treatment period. Similarly, the control group increased the number of followed accounts, from 13.98 to 22.68. Due to the API-driven challenges of the followee collection and the statistically insignificant increase of our treatment groups relative to the control, we do not put too much emphasis on this finding.

Centrally, there was an increase in users' liking of news content, namely the post- relative to pre-treatment proportion of likes on posts coming from one of the 5,341 news organizations relative to all likes. In particular, the users in the female treatment increased in their liking of news content from 0.79% to 0.93% (a 0.14% increase), compared to users in the control group who increased from 0.82% to 0.85% (a 0.03% increase), a statistically significant difference (p=0.02). Users in the male treatment, however, increased their percentage of news likes by the same amount as the control (0.03% increase, p=0.55), suggesting that this effect emerged in the female treatment group only.

Furthermore, there was an increase in the liking and (re)tweeting of political content across all groups, with the largest increase again in the female treatment group (control: from 11.85% to 12.23%, female: from 11.81% to 12.18%, male: from 11.93% to 12.05%). However, relative to the control, these differences were not statistically significant for either treatment group (female p=0.12).

For the other measured variables, i.e., tweets or retweets which mention media organizations and tweets or retweets which contain political content, post-treatment levels were marginally lower in all treatment groups, with no differences being statistically significant relative to the control group.

7. Re-Weighting

As the profile of users who were ultimately treated was not randomly distributed (accounts more likely to tweet were more likely to tweet a keyword-matching tweet which would receive a treatment), a re-weighting of the control (all assigned users) relative to the treatment groups (partial assigned users dependent on treatment status) was required prior to measuring treatment effects. The re-weighting of groups was done using entropy balancing, using the "WeightIt" package in R. Weighting was conducted based on the same account-level characteristics used for randomized user assignment, collected prior to treatment. Below we show the adjusted means for each treatment-control combination based on the "intention-to-treat" (ITT) and "Treated" samples.

Combination	Metric	Adj. Mean Diff. (ITT)	Adj. Mean Diff. (Treated)
**Control - Male**	Favourites count	0	0.0022
**Control - Male**	Statuses count	0	0.0129
**Control - Male**	Friends count	0	0.0030
**Control - Male**	Followers count	0	0.0057
**Control - Female**	Favourites count	0	0.0022
**Control - Female**	Statuses count	0	0.0073
**Control - Female**	Friends count	0	0.0028
**Control - Female**	Followers count	0	0.0046

Table S8. Balance Between Groups After Entropy Matching, Adjusted Mean Difference on Variable.

As can be seen, all variables are almost perfectly balanced in every control-treatment combination. As groups were already balanced on these variables prior to treatment, re-weighting is effectively redundant in the ITT group and precisely perfect weighting is achieved between groups. In the Treated sample, balance is close to perfect and well below the standard threshold of 0.1 absolute adjusted mean difference.

8. Full Regression Models

Full Regression with Entropy Balancing (Intention to Treat)

Treatment	Following	News (Re)tweets	News Likes	Political Tweets	Political Likes
Female	0.0518	-0.02305	0.0104	-0.00885	-0.02738
	(0.0278)	(0.0169)	(0.0176)	(0.0169)	(0.0175)
Male	0.0647	-0.00461	0.0384	0.02559	-0.00719
	(0.0277)	(0.0169)	(0.0176)	(0.0169)	(0.0175)
Combined	0.00604	-0.0138	0.0244	0.00841	-0.0173
	(0.02)	(0.0147)	(0.0152)	(0.0147)	(0.0152)
Observations	11,244	20,966	19,507	20,966	19,507

Note: Estimated using pairwise G-Computation after entropy balancing. Robust standard errors.
Central variables are standardized with a mean of 0 and a Standard deviation of 1.

Table S9. Full Regression with Entropy Balancing (Intention to Treat)

Full Regression with Entropy Balancing (Treated)

Treatment	Following	News (Re)tweets	News Likes	Political Tweets	Political Likes
Female	0.07018	-0.0034	0.03998	0.02465	-0.0000817
	(0.0308)	(0.0183)	(0.0191)	(0.0186)	(0.0192)
Male	0.06399	-0.0201	0.00377	-0.00853	-0.02077
	(0.0308)	(0.0184)	(0.0193)	(0.0188)	(0.0194)
Combined	0.03	0.0131	0.0217	0.0124	-0.0114
	(0.0244)	(0.0176)	(0.0184)	(0.0178)	(0.0184)
Observations	8,891	16,895	15,764	15,764	15,764

Note: Estimated using pairwise G-Computation after entropy balancing. Robust standard errors.
Central variables are standardized with a mean of 0 and a Standard deviation of 1.

Table S10. Full Regression with Entropy Balancing (Treated)

High Political Interest Regression with Entropy Balancing (Intention to Treat)

Treatment	Following	News (Re)tweets	News Likes	Political Tweets	Political Likes
Female	0.02472	0.0049	0.0550	0.0175	0.0117
	(0.0307)	(0.022)	(0.023)	(0.0221)	(0.0230)
Male	-0.00295	-0.0216	0.0306	-0.0210	-0.0234
	(0.0307)	(0.022)	(0.023)	(0.0220)	(0.0229)
Observations	6,416	12,358	11,443	12,358	11,443

Note: Estimated using pairwise G-Computation after entropy balancing. Robust standard errors.

Table S11. High Political Interest Regression with Entropy Balancing (Intention to Treat)

Low Political Interest Regression with Entropy Balancing (Intention to Treat)

Treatment	Following	News (Re)tweets	News Likes	Political Tweets	Political Likes
Female	-0.00847	-0.0178	0.0211	0.0701	-0.0154
	(0.0377)	(0.0273)	(0.028)	(0.0272)	(0.0281)
Male	0.03964	-0.0362	-0.0323	0.0320	-0.0295
	(0.0378)	(0.0273)	(0.028)	(0.0272)	(0.0281)
Observations	4,167	8,054	7,548	8,054	7,548

Note: Estimated using pairwise G-Computation after entropy balancing. Robust standard errors.

Table S12. Low Political Interest Regression with Entropy Balancing (Intention to Treat)

High Political Interest Regression with Entropy Balancing (Treated)

Treatment	Following	News (Re)tweets	News Likes	Political Tweets	Political Likes
Female	0.05316	-0.00105	0.0552	0.00865	0.0166
	(0.0337)	(0.0239)	(0.0250)	(0.0242)	(0.0251)
Male	0.00021	-0.02665	0.0180	-0.03770	-0.0122
	(0.0338)	(0.0240)	(0.0251)	(0.0243)	(0.0252)
Observations	5,163	10,010	9,306	9,306	9,306

Note: Estimated using pairwise G-Computation after entropy balancing. Robust standard errors.

Table S13. High Political Interest Regression with Entropy Balancing (Treated)

Low Political Interest Regression with Entropy Balancing (Treated)

Treatment	Following	News (Re)tweets	News Likes	Political Tweets	Political Likes
Female	0.00655	-0.04061	0.0173	0.0382	-0.03258
	(0.0408)	(0.0295)	(0.0304)	(0.0295)	(0.0302)
Male	0.03310	-0.03758	-0.0285	0.0415	-0.03725
	(0.0411)	(0.0298)	(0.0307)	(0.0298)	(0.0305)
Observations	3,477	6,715	6,303	6,303	6,303

Note: Estimated using pairwise G-Computation after entropy balancing. Robust standard errors.

Table S14. Low Political Interest Regression with Entropy Balancing (Treated)

9. Alternate Regression Models (News Account Following Models)

In the main paper we excluded certain users from the news account following models who changed their total followers by an unusually large amount over the experimental time period. Here we test the main effects by adjusting the values of the manual user exclusion criteria. In general, excessive increases in the number of followers highlight unrepresentative user behavior, independent of treatment exposure (e.g., users cleaning their accounts or engaging in large-scale account following sprees), indicating that any news accounts removed or captured in these behaviors may be incidental. While excluding the top and bottom 10 percentiles of the account following change distribution help systematically remove the most extreme cases, the addition of a manual cutoff is arguably less systematic. We therefore present the results comparing the threshold of 200 accounts (a relatively generous cutoff for a two week treatment time period), with a more loose cutoff of 500 accounts, as well as with models where no manual cutoff is included.

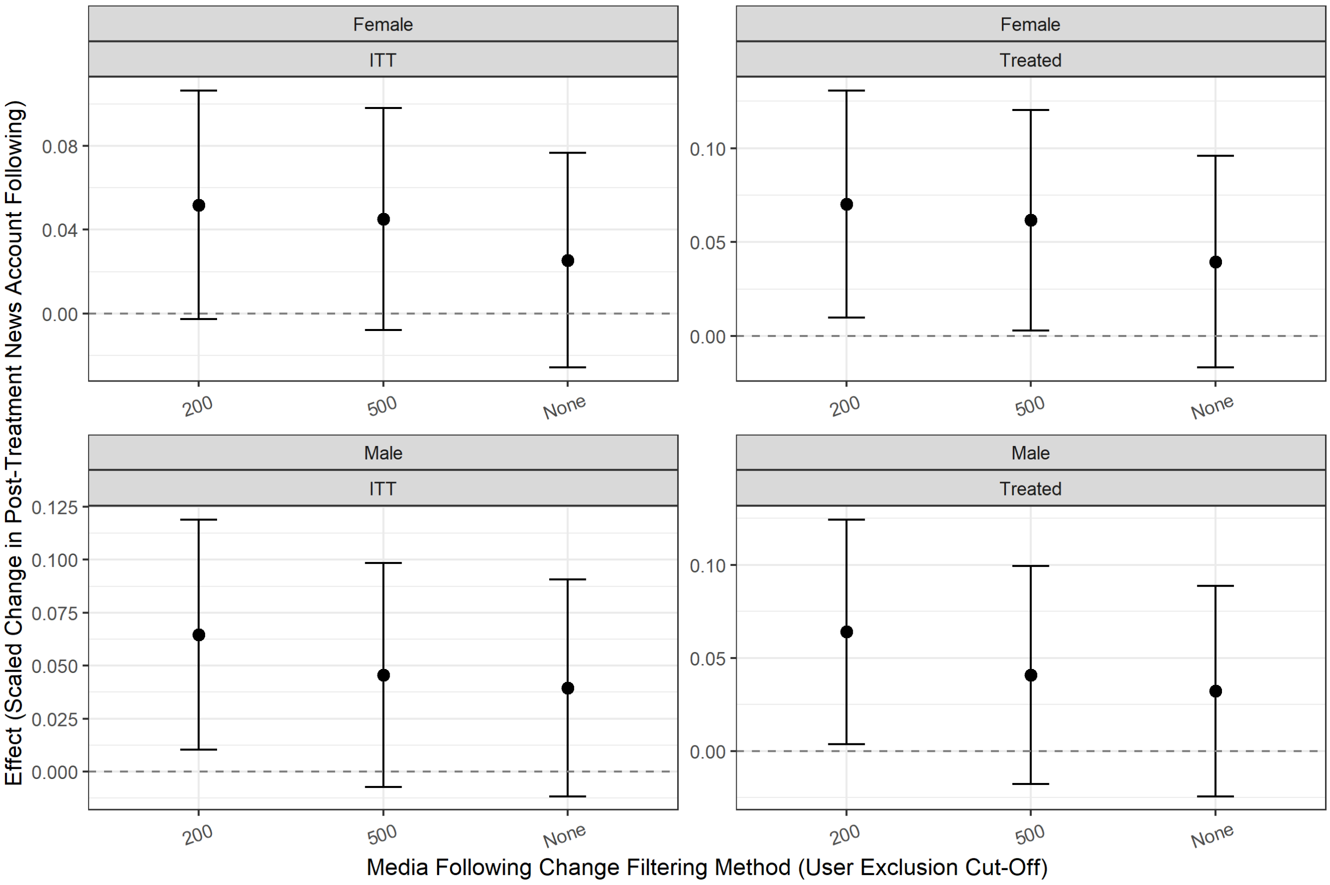

Fig. S1. News Following Treatment Effects Divided by Bot Gender and Exclusion Criteria: Coefficient estimates and 95% confidence intervals for G-computation after entropy balancing regression models with robust standard errors. 200 refers to an exclusion of all users who increased their news media following by more than 200 accounts in the post-treatment collection, 500 refers to an exclusion of 500 or more, none contains no count exclusion criteria. All models still retain a baseline exclusion criteria of a more than -20% and less than 50% change.

A. Suggested News Account Following Models. We also test whether users followed the specific handles in our pool of suggested news media accounts. The Male and Female treatment groups do see a greater increase in the following of these accounts in the post-treatment measurement. However, repeating the entropy balancing and regression modelling on this measure does not show a significant difference.

Treatment	Post-Treatment Change	ITT (P-value)	Treated (P-Value)
Control	4.27	-	-
Male	4.42	0.211	0.139
Female	4.38	0.370	0.181

Table S15. Post-Treatment Change in Media Accounts Followed (Select Pool of Suggested Accounts). Raw post-treatment change shown plus p-values from regression with entropy balancing on ITT and Treated user pools, relative to the control group.

10. Exploratory Analysis by Topic

We additionally explore whether the treatment effects differed by topic category by running separate models for users who—during the treatment period—tweeted about entertainment versus lifestyle versus sports. We perform topic-wise classification of our users into these 3 categories by doing a combination of keyword matching and BERT-based embedding matching on all of the tweets we collected for each user in our 2 week period. Whichever category received the most tweets by the user was classified as the category for that user. The final user category percentages via this method was 61.30% Sports, 30.67% Entertainment and 8.03% Lifestyle.

As shown in Figure S2, the effects are very limited, again due to the substantially smaller sub-sample sizes, especially among the treated groups. The only statistically significant effect emerges among users who tweeted about sports. There, we find that the users in the female bot treatment group increased their liking of news media content relative to the control in both the ITT and the treated models. We also see a significant result for the male treatment among the users tweeting about entertainment, finding that comments from a male bot increased the liking of news media content. This effect, however, becomes insignificant once we focus on the treated group.

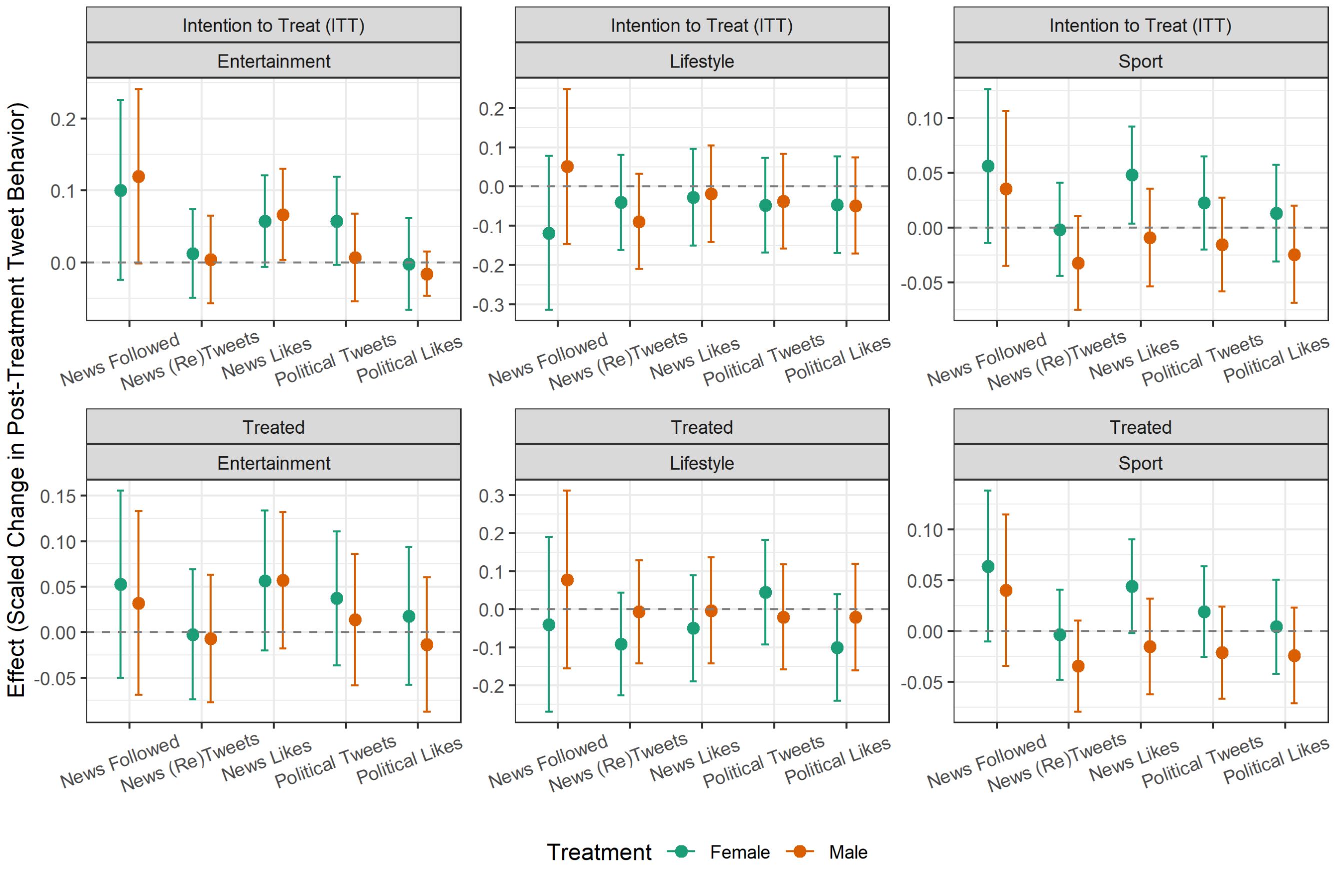

Fig. S2. Main Treatment Effects Divided by Users' Primary Topic of Twitter Activity: Coefficient estimates and 95% confidence intervals for G-computation after entropy balancing regression models with robust standard errors. Dependent variables taken as the difference between pre- and post-treatment individual user measures. News media accounts followed measured as a count, news media tweets and likes and political tweets and likes measured as percentages.

Although whatever significant effects mostly emerged among the users who were tweeting about sports, this is likely due to the fact that sports users were the majority of our sample. We thus caution against putting too much leverage on these findings because the sample sizes became substantially smaller when sub-divided into the three topic categories.

References

1. S Wojcik, A Hughes, Sizing up twitter users (https://www.pewresearch.org/internet/2019/04/24/sizing-up-twitter-users/) (2019).
2. D Freelon, Geostring (https://github.com/dfreelon/geostring) (2023).
3. M Honnibal, I Montani, S Van Landeghem, A Boyd, spaCy: Industrial-strength Natural Language Processing in Python (2020).
4. M Sayyadiharikandeh, O Varol, KC Yang, A Flammini, F Menczer, Detection of novel social bots by ensembles of specialized classifiers in *Proceedings of the 29th ACM International Conference on Information & Knowledge Management*, CIKM '20. (Association for Computing Machinery, New York, NY, USA), p. 2725–2732 (2020).
5. A Chhabra, Political classifier (https://github.com/anshuman23/political_classifier) (2023).
6. Mail.com (https://www.mail.com/) (2023).
7. textverified.com (https://www.textverified.com/) (2023).
8. This person doesn't exist (https://this-person-does-not-exist.com/en) (2023).
9. Y Zhang, et al., Dialogpt: Large-scale generative pre-training for conversational response generation. *arXiv preprint arXiv:1911.00536* (2019).
10. A Radford, et al., Language models are unsupervised multitask learners. *OpenAI blog* **1**, 9 (2019).
11. M Nye, M Tessler, J Tenenbaum, BM Lake, Improving coherence and consistency in neural sequence models with dual-system, neuro-symbolic reasoning. *Adv. Neural Inf. Process. Syst.* **34** (2021).
12. C Donahue, M Lee, P Liang, Enabling language models to fill in the blanks. *arXiv preprint arXiv:2005.05339* (2020).
13. GPT-2 Medium Topic News (https://huggingface.co/ktrapeznikov/gpt2-medium-topic-news) (2022) Accessed: 2022-04-17.
14. V Otero, Ad fontes media's first multi-analyst content analysis ratings project (2019).
15. M Wojcieszak, et al., Non-news websites expose people to more political content than news websites: Evidence from browsing data in three countries. *Polit. Commun.* **41**, 129–151 (2024).
16. Y Liu, et al., Roberta: A robustly optimized bert pretraining approach. *arXiv preprint arXiv:1907.11692* (2019).
17. J Devlin, MW Chang, K Lee, K Toutanova, Bert: Pre-training of deep bidirectional transformers for language understanding. *arXiv preprint arXiv:1810.04805* (2018).
18. Tweepy documentation (https://docs.tweepy.org/en/stable/api.html) (2023).
19. D Loureiro, F Barbieri, L Neves, LE Anke, J Camacho-Collados, Timelms: Diachronic language models from twitter (2022).
20. F Barbieri, J Camacho-Collados, L Neves, L Espinosa-Anke, Tweeteval: Unified benchmark and comparative evaluation for tweet classification (2020).
